# Supplementary material for: Identification of predicate creep under the 510(k) process: A case study of a robotic surgical device
Source: PLoS One. 2023 Mar 28;18(3):e0283442. doi: 10.1371/journal.pone.0283442 (PMC10047502; doi:10.1371/journal.pone.0283442)
Supplement: Attachment — Submitted filename: Response to Reviewer 2nd revision.docx [file pone.0283442.s002.docx]

To the Journal Editor and Reviewers:

*Thank you for giving us the opportunity to revise this paper. We are particularly grateful for the attention to detail of the reviewer, and willingness to look through a 103 page document! Below you will find a detailed response to yours and the reviewers’ comments. Our responses are in italics.*

Substantive:

-The updated focus on product codes is nicely done, and a good addition to the literature. The revised Figure 2 captures this quite well in particular. Kudos.

*Thank you again for your suggestion to change the focus of the paper. We agree that it improved the paper significantly.*

-In the discussion, I think the authors should be clear that the limitations of their work are not simply to a specific type of device (Robotic Surgery devices), but also to a device category which is highly consolidated (limited competition – which means there are fewer available predicates that likely can be cited) and is by definition composed of multiple different devices (a similar issue for devices like orthopedic implants, but perhaps less of an issue for devices such as respiratory machines or endovascular catheters)

You are correct that this is a limit. We have added the following statement.

*Moreover, given that for many years there was little or no competition to the Da Vinci Surgical Systems, we chose a device category for which there are likely to be fewer products available to cite as predicates than for products with higher levels of market competition.*

-Introduction (page 18): the paragraph starting with “FDA has responded to these concerns…” could be deleted from the introduction. Some of this content is already in the discussion, and that is a more appropriate place for it. This change would also help shorten the introduction.

*We agree and have taken out this paragraph*

-Literature Review (page 23): Please revise this sentence: “The uniqueness of the device as an emerging technology makes it representative of the many challenges…”. It does not follow that a “unique” example can also be “representative”.

*You are absolutely right, and the sentence was also a bit repetitive. We removed this sentence.*

-When tracking product code divergence, did the authors also confirm that the change in product code was a decision by the manufacture, and not because of a reclassification order by FDA? As the latter would mean that it wasn’t so much an instance of “predicate creep” as it was updated regulation

*We did not determine if the product classification code was made by the manufacturer or the FDA, or if it was driven by an administrative need or a technical feature. We now mention this in the limitations section. “Lastly, we did not determine if the product classification code was made by the manufacturer or the FDA, or if it was driven by an administrative reason or a technical feature.”*

-Data Analysis (page 29): “This illustrates how larger jumps in the technological complexity of devices new devices can occur through the 510(k) process” – great summary statement. Note that there seems to be a typo, with “devices” repeated twice. Otherwise, this well captures the contributions of this study.

*Thank you for catching this. It has been corrected.*

-Discussion (page 32): The sentence “…to reduce barriers to bringing new medical devices” is not quite correct (that is perhaps a summation of the “Least Burdensome Principle”). The purpose of 510(k) is to facilitate incremental innovation in medical device development; not just reduce barriers. Please correct.

*We have rewritten the sentence as “Given the purpose of the 510(k) process is to encourage incremental innovation in the medical device market by reducing regulatory barriers…..”*

-Discussion (page 32): The sentence “..pre…”. “Guarantee” may not be appropriate here. The IOM report on 510(k) is clear that 510(k) clearance does not demonstrate safety. Would consider revising.

*Thus, predicate creep in cases where the new device can be reasonably assured safe based on available scientific evidence, is beneficial to companies, patients, and regulators.*

-Discussion (page 33): “We were also able to see the absorption of a primary predicate device function into a secondary system function” – a really important interesting insight. Nicely done.\

*Thank you*

-Limitations (page 35): In the sentence “scope of this research to robotic surgical devices”; it also may be useful to point about the fact about consolidation/monopolization of this space, which makes it harder to generalize to other product codes where there may be more device makers (and hence, more predicates)

We have added this sentence:

*Moreover, given that for many years there was little or no competition to the Da Vinci Surgical Systems, we chose a device category for which there are likely to be fewer products available to cite as predicates than for products with higher levels of market competition.*

-Implications for Research (Page 36): the point about patents is really interesting. Of note, there’s no good resource for this for device (e.g., there’s no equivalent of the FDA Orange Book for generic medicines as there is for devices).

*That is true. We have noticed this when comparing research approaches with our research group’s work on pharmaceuticals*

-Implications for Policy (Page 38): The discussion about Step and Leap devices is interesting; I think the authors could provide more weight and clarity to this section by inverting the order of the current organization. First, highlight what FDA has already done, and then offer recommendations; bounces around a little too much right now.

*Thank you for this suggestion. We have changed the order and it does make more sense now.*

Stylistic

-The figures appear a bit blurry in my version of the PDF. Assuming Editors will work with the authors to ensure the figures in the final version of the paper are of a higher resolution

*We agree. We will have to talk to the journal about this. The figures in our document are clear - but they change when uploaded into the system.*

-At several points in the paper, the authors use the word “approved” (for example, page 21 of the Literature “These predicates can have also been approved…”). 510(k) does not provide “approvals”; the appropriate term is either “clearance” or “authorization”. Please revise all references throughout

*Very good point. You are correct. We have changed all “approved” to “cleared”*

-Throughout the article, the authors alternate from “Da Vinci” and “Da Vinci Si”. Please use just one consistent name.

*Thank you for this suggestion. Except for cases where we are talking about the Da Vinci systems as a whole or another model, we have changed all mentions to* Da Vinci Si

-In the Implications for Future Research section, consider revising instances of “should” to “could” when discussing recommendations for FDA

*We have made this change*

-Abstract (page 17): last sentence has a grammatical issue (“…discuss implications of this method finding for research and policy”). Perhaps the authors meant “methodological”?

*We have changed this to read “We find that there is evidence of predicate creep using our method, and discuss implications of this method for research and policy.”*

-Literature Review (page 20): in the sentence “Substantially equivalence, however, proves only…”); I’d advise the authors to find an alternative word for “proves”. SE determinations are less of a tried-and-true fact, and more of a reflection of a regulatory assessment

*You are absolutely correct. We have changed this to read “Substantial equivalence, however, only supports an assessment that the device introduces no new safety hazards and functions at least as effectively as the predicate device.”*

-Literature Review (page 20): For the sentence “It is not uncommon for a device to cite a recalled predicate”, consider citing the new study by Everhart and colleagues in JAMA 2023 which estimates the prevalence of this phenomenon to be ~5% of all 510(k) devices

*Thank you for pointing out this article. I added this and another article in that same issue as references.*

-Literature Review (page 21): Seems like there’s a typo in this sentence (“…systems, and found that (a cycle.7%) 510(k) clearances did not..”). Please fix.

*This has been fixed*

-Literature Review (page 23): The authors use “database” as one word and “data bases” as two words in on this page. Please stick to just one word.

*We have made this correction.*

-Literature Review (page 22): When discussing FOIA requests, could reference FDA’s website for this process, which provides timelines for how long this takes. Could also reference studies from the literature showing the limitations of what information FDA is willing to redact

*We have added a reference to the FDA time estimate to our discussion of FOIA, which is now in the conclusion. Since the issue of redacted data was not discussed in the paper, we did not add references regarding this issue. It now reads “*We did consider requesting additional data through FOIA, but decided against this route given that it would add a significant amount of time to the data gathering, with an FDA estimate of 18 to 24 months [24], and there was a precedent in prior research to rely primarily on information provided in the FDA website [11].

-Implications for Policy (page 38): “…”step” devices, which are submit and approved…”. “Submit” should be “Submitted”.

*This has been corrected*

*Thank you again for your detailed comments. It has significantly improved the quality of this paper.*
